# Reduced TREM2 activation in microglia of patients with Alzheimer's disease

**DOI:** 10.1002/2211-5463.13300

**Published:** 2021-09-28

**Authors:** Yuumi Okuzono, Hiroyuki Sakuma, Shuuichi Miyakawa, Masataka Ifuku, Jonghun Lee, Debashree Das, Antara Banerjee, Yang Zhao, Koji Yamamoto, Tatsuya Ando, Shuji Sato

**Affiliations:** ^1^ Immune Cell Engineered Therapeutics Research, Takeda Pharmaceutical Company Limited Fujisawa Japan; ^2^ Neuroscience Drug Discovery Unit Research, Takeda Pharmaceutical Company Limited Fujisawa Japan; ^3^ Computational Biology Research, Takeda Pharmaceutical Company Limited Fujisawa Japan; ^4^ Early Target Discovery Research, Takeda California, Inc. San Diego CA USA; ^5^ GI Immunology Research, Takeda California, Inc. San Diego CA USA

**Keywords:** Alzheimer's disease, differential expression analysis, gene set enrichment analysis, microglia, single‐nucleus RNA sequencing, TREM2

## Abstract

Loss‐of‐function variants of triggering receptor expressed on myeloid cells 2 (TREM2) increase the risk of developing Alzheimer's disease (AD). The mechanism through which TREM2 contributes to the disease (TREM2 activation vs inactivation) is largely unknown. Here, we analyzed changes in a gene set downstream of TREM2 to determine whether TREM2 signaling is modified by AD progression. We generated an anti‐human TREM2 agonistic antibody and defined TREM2 activation in terms of the downstream expression changes induced by this antibody in microglia developed from human induced pluripotent stem cells (iPSC). Differentially expressed genes (DEGs) following TREM2 activation were compared with the gene set extracted from microglial single nuclear RNA sequencing data of patients with AD, using gene set enrichment analysis. We isolated an anti‐TREM2‐specific agonistic antibody, Hyb87, from anti‐human TREM2 antibodies generated using binding and agonism assays, which helped us identify 300 upregulated and 251 downregulated DEGs. Pathway enrichment analysis suggested that TREM2 activation may be associated with Th2‐related pathways. TREM2 activation was lower in AD microglia than in microglia from healthy subjects or patients with mild cognitive impairment. TREM2 activation also showed a significant negative correlation with disease progression. Pathway enrichment analysis of DEGs controlled by TREM2 activity indicated that TREM2 activation in AD may lead to anti‐apoptotic signaling, immune response, and cytoskeletal changes in the microglia. We showed that TREM2 activation decreases with AD progression, in support of a protective role of TREM2 activation in AD. In addition, the agonistic anti‐TREM2 antibody can be used to identify TREM2 activation state in AD microglia.

## Graphical abstract

We conducted gene set enrichment analysis between differentially expressed genes following triggering receptor expressed on myeloid cells 2 (TREM2) activation and the gene set extracted from microglia of patients with Alzheimer's disease. The analysis demonstrated that TREM2 activation was lower in Alzheimer's disease microglia than in microglia from healthy subjects. We are the first to show TREM2 activation status in diseased patients.

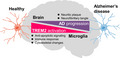

**Abbreviations**
Ab: antibody
AD: Alzheimer's disease
BLI: biolayer interferometry
CERAD: Consortium to Establish a Registry for Alzheimer's Disease
cogdx: clinical consensus diagnosis of cognitive status at the time of death
CSF: cerebrospinal fluid
DEG: differentially expressed gene
FCM: flow cytometry
FDR: false discovery rate
GSEA: gene set enrichment analysis
HC: healthy control
HPC: hematopoietic progenitor cell
iHPC: iPSC-derived hematopoietic progenitor cell
iMG: iPS-derivedmicroglia-like cell
iPS: induced pluripotent stem cell
KO: knockout
mAb: monoclonal antibody
MCI: mild cognitive impairment
MMSE: Mini-Mental State Examination
NES: normalized enrichment scores
NFAT: nuclear factor of activated T cells
PC: principal component
PCA: principal component analysis
ROSMAP: Religious Orders Study and Rush Memory and Aging Project
signed *P* value: −log_10_ *P* value with the sign of the log-scaled fold change
snRNA-seq: single-nucleus RNA-seq
sTREM2: soluble form of TREM2
TREM2: triggering receptor expressed on myeloid cells 2

Alzheimer's disease (AD), the most common cause of dementia, is characterized by cognitive decline and memory deficits. According to the World Health Organization, more than 30 million people suffer from AD worldwide. Despite the high prevalence of the disease, disease‐modifying agents that can slow or stop neurodegeneration are not known and the unmet therapeutic requirements for AD are immense. Currently, the available therapies for AD, such as acetylcholinesterase inhibitors and/or a noncompetitive *N*‐methyl‐d‐aspartate receptor antagonist, provide only symptomatic relief and do not restrict or halt disease progression [1].

Human genetic studies have indicated that loss of function of the triggering receptor expressed on myeloid cells 2 (TREM2) correlates with increased risk of AD [2, 3]. Furthermore, as the ε4 allele of apolipoprotein E—a TREM2 ligand—is the dominant genetic risk factor for late‐onset AD (LOAD) [4], and TYROBP—a TREM2 adaptor protein—has been identified as the key regulator of LOAD using an integrative network‐based approach [5], TREM2 has emerged as an important signaling molecule in AD. TREM2 risk variants are associated with neuropathology [3]. The R47H mutation—a rare TREM2 loss‐of‐function variant—impairs TREM2 ligand recognition [6, 7, 8, 9] and alters its glycosylation pattern, leading to its instability on the cell surface and degradation in the lysosomes [10, 11] and attenuation of downstream signaling [6, 9]. In AD and mild cognitive impairment (MCI), TREM2 R47H carriers exhibit substantial gray matter loss in the orbitofrontal cortex and the anterior cingulate cortex, with relative sparing of the parietal lobes [12]. Furthermore, R47H carriers with LOAD have increased neuritic plaque and neurofibrillary tangle densities [13]. Even cognitively normal elderly people with R47H are known to have poorer cognitive function than noncarriers [2]. A brain‐imaging volumetric study of individuals with a risk allele near R47H revealed that the mutation carriers lose brain volume at a significantly faster rate than the noncarriers [14]. These findings prompted the scientific community to verify whether TREM2 activation could be a therapeutic option for AD [15] and ameliorate AD disease pathology in several mouse models. TREM2 activation of microglia reduces extracellular amyloid plaques and increases the number of microglia surrounding the plaques in multiple mouse AD models, including the APP/PS1 model with lentiviral TREM2 overexpression [16] and the 5xFAD model with microglial TREM2 overexpression [17]. Furthermore, TREM2 overexpression attenuates neuronal loss and promotes behavioral improvement in 5xFAD and APP/PS1 models [16, 17]. Similar findings have been reported in studies based on mutant tau mouse models. TREM2 overexpression reduces tau phosphorylation and inflammatory cytokine production and improves neuronal survival and spatial memory function in the tau P301S mouse model [18]. Recent studies have reported the protective functions of TREM2 in AD preclinical models using anti‐TREM2 antibodies (Abs) [19, 20]. These studies have highlighted the importance of TREM2 in AD onset and progression and indicated that TREM2 activation may provide a therapeutic option for patients with AD.

Triggering receptor expressed on myeloid cells 2 expression in AD brain tissues has been examined in several reports. However, findings are divided, whether TREM2 expression is altered in the AD tissues. One study found that TREM2 mRNA expression increased in the hippocampus of patients with AD [21]. However, other studies have shown downregulation or no significant changes in TREM2 expression in the hippocampus samples from AD patients at protein and/or mRNA levels [22, 23, 24]. TREM2 expression was higher in the temporal cortex of patients with AD than that in the corresponding tissues of non‐AD donors at mRNA and/or protein levels [25, 26]. In the frontal cortex, TREM2 expression was significantly elevated at the protein level in AD patients, but not at the mRNA level. A recent report indicated that TREM2 is upregulated in the microglia of the dorsolateral prefrontal cortexes of patients with AD [27]. Considering the supporting genetic evidence that TREM2 loss‐of‐function mutation increases the risk of AD, it is highly important to decipher whether TREM2 upregulation is associated with enhanced TREM2 signaling, thereby retarding the disease progression. Another notable feature of TREM2 is the presence of a soluble form (sTREM2). The membrane protein TREM2 is cleaved by the proteases ADAM10 and ADAM17 to release sTREM2 in response to various stimuli [28, 29]. sTREM2 has been used as a microglial activation marker, and its levels were found to be high in the cerebrospinal fluid (CSF) of patients with AD; moreover, it correlates positively with total and phosphorylated tau levels [30, 31, 32]. Although reports have shown that sTREM2 is produced following TREM2 overexpression and that sTREM2 levels depend on TYROBP [28, 33], whether sTREM2 levels reflect TREM2 activation in the disease remains unclear. sTREM2 levels in AD may indicate TREM2 upregulation, or change in activity, or expression of the responsible proteases. However, considering that the rarely occurring R47H loss‐of‐function mutant produces sTREM2 more efficiently than the common variant of TREM2 [34], sTREM2 levels may not necessarily reflect the activation status of TREM2. Therefore, further studies are necessary to understand TREM2 activation status in the AD brain and elucidate the mechanism through which changes in TREM2 signaling contribute to AD.

In this study, we used a gene set acting downstream of TREM2 and aimed to investigate whether the TREM2 signal is modulated with AD progression. We further analyzed the potential of using TREM2 activation as a therapeutic option for AD.

## Materials and methods

### Generation of anti‐TREM2 monoclonal antibody

All animal‐related research protocols used in this study were approved by the Takeda Institutional Animal Care and Use Committee. Five CD2F1 mice (6 weeks old, female; The Jackson Laboratory, Bar Harbor, ME, USA) and five Trianni IgH kappa mice (6 weeks old, female; Trianni, Inc., San Francisco, CA, USA) were housed in the animal facilities of Takeda California and maintained under regular specific pathogen‐free conditions. Following 1 week of acclimatization, the mice were immunized subcutaneously into the hock with 5 µg human TREM2‐Fc (R&D Systems, Minneapolis, MN, USA) emulsified with TiterMax Gold adjuvant (TiterMax, Norcross, GA, USA) as part of the first immunization. For the second through the ninth immunizations, the mice were injected subcutaneously into the hock twice weekly with 3 μg human TREM2‐Fc using ODN‐1826 (InvivoGen, San Diego, CA, USA) and alum adjuvant. On the day of the third immunization, the mice were intraperitoneally injected with 100 μg anti‐mCD40 Ab (BioXcell, West Lebanon, NH, USA). Three days after the final boost, the popliteal and inguinal lymph nodes were isolated from each mouse, and the lymphocytes were fused with P3U1 mouse myeloma cells (ATCC, Manassas, VA, USA) in a 2 : 1 ratio and subjected to electrofusion using a Legacy ECM 2001 electrocell fusion and electroporation system (BTX, Holliston, MA, USA) to generate hybridomas. The fused cells were seeded in a semisolid selective culture media containing hypoxanthine–aminopterin–thymidine, and the grown hybridoma clones were manually transferred to 96‐well plates. Hybridomas secreting anti‐TREM2 monoclonal antibody (mAbs) were screened using ELISA. First, the human TREM2‐Fc protein was coated on a 96‐well ELISA plate and then incubated with hybridoma culture supernatant and goat anti‐mouse IgG (H + L) conjugated with horseradish peroxidase (HRP; Jackson ImmunoResearch Laboratories, Inc., West Grove, PA, USA). The plates were washed with PBS containing 0.05% Tween‐20 after each incubation step. The TMB HRP substrate (Bio‐Rad, Hercules, CA, USA) was added to each well and incubated for 5 min. The reaction was stopped using 1 m H_2_SO_4_, and the absorbance at 450 nm was measured using an EnVision plate reader (PerkinElmer, Waltham, MA, USA).

The Hyb87 hybridomas were expanded in Ab expression medium, which was a mixture of Iscove's modified Eagle medium and Ham's F‐12 nutrient medium (FujiFilm Wako Pure Chemical, Osaka, Japan) supplemented with MEM nonessential amino acid solution, sodium pyruvate, l‐alanyl‐l‐glutamine, 100 U·mL^−1^ of penicillin and streptomycin (FujiFilm Wako Pure Chemical), and 10% ultra‐low IgG fetal bovine serum (Thermo Fisher Scientific, Waltham, MA, USA). Hyb87 was purified from the culture supernatant using Ab‐Capcher ExTra (ProteNova, Higashikagawa, Japan).

### Determination of Ab binding to TREM2 on cells

#### Expi293 cells

We tested the specificity of Hyb87 binding to TREM2 using flow cytometry (FCM). Briefly, a vector expressing human TREM2 and the NeoFection reagent (Astec Co. Ltd., Kasuya, Japan) were mixed at 1 : 1 ratio in OptiMEM I (Thermo Fisher Scientific). After 15 min, the mixture was added to Expi293 cells (Thermo Fisher Scientific) at a density of 1 × 10^6^ cells·mL^−1^. One day after transfection, the cells were reacted with Hyb87 or control IgG (R&D Systems), followed by incubation with anti‐mouse IgG‐Alexa Fluor 488 (Thermo Fisher Scientific). Cell surface fluorescence was detected using BD Accuri C6 Plus (BD Biosciences, San Jose, CA, USA) and analyzed using the FlowJo software (Becton Dickinson, Ashland, OR, USA).

#### THP‐1 cells

Similarly, we tested the specificity of Hyb87 using the human monocytic THP‐1 cells (ATCC) and *TREM2* knockout (KO) THP‐1 cells following the Expi293 procedures. *TREM2* KO THP‐1 cells were established in our laboratory as mentioned below. The Fc receptor blocking reagent (BD Biosciences) was used during Ab incubation with THP‐1 cells or *TREM2* KO THP‐1 cells. In brief, duplex RNA was prepared by annealing equimolar amounts of crRNA (5′‐ACCCAGGGTATCGTCTGTGATGG‐3′ or 5′‐CACAGTGTTCCAGGGCGTGGCGG‐3′) and tracrRNA at 95 °C for 5 min and cooling to room temperature. To form the ribonucleoprotein (RNP) complex, Cas9 was added to the duplex RNA and incubated for 15 min at room temperature. The THP‐1 cells were simultaneously transfected with both complexes using the Neon transfection system (Thermo Fisher Scientific) and seeded at a density of 2 × 10^5^ cells·well^−1^ in THP‐1 culture medium supplemented with 1 μm RS‐1 and 0.1 μm SCR7 (Xcess Biosciences, Chicago, IL, USA) in a 24‐well plate. Seven days following the transfection, the cells were subcloned using limiting dilution method at 0.3 or 1 cell·well^−1^. Genomic DNA was extracted from the outgrown cells using the SimplePrep reagent for DNA (Takara Bio, Kusatsu, Japan) and sequenced using the BigDye Terminator v3.1 cycle sequencing kit (Thermo Fisher Scientific). We selected clone 17 with a frameshift mutation in the *TREM2* exon as *TREM2* KO THP‐1 and used it for FCM.

### Determination of Ab affinity

The kinetic analysis of the affinity of Ab to TREM2 was performed using biolayer interferometry (BLI) with an Octet Red96e system (Molecular Devices, Sunnyvale, CA, USA). First, Hyb87 was captured on anti‐mouse Fc Octet biosensors (Molecular Devices) for 120 s. The biosensors were reacted with serially diluted recombinant human TREM2‐His (R&D Systems) for 120 s, followed by dissociation in PBS for 180 s. The kinetics of the Ab was analyzed with a sensorgram aligned at the beginning of the association step after background subtraction. The sensorgram was analyzed using a 1 : 1 Langmuir fitting model.

### Nuclear factor of activated T cells assay

Triggering receptor expressed on myeloid cells 2 activation was detected as described below. Hyb87 or control IgG (10 μg·mL^−1^) was immobilized on a 96‐well plate (Corning, New York, NY, USA, #3912) overnight at 4 °C. One million THP‐1 or *TREM2* KO THP‐1 cells were transfected with 5 μg of the luciferase reporter plasmid driven by the nuclear factor of activated T cells (NFAT) response element using the Neon transfection system (Thermo Fisher Scientific) according to the manufacturer's instructions. In total, 30 000 transfected cells were seeded on the Ab‐immobilized plate. After 3 h, luciferase activity was detected with NanoGlo reagent (Promega, Madison, WI, USA) using an EnVision plate reader (PerkinElmer).

### Treatment of induced pluripotent stem cell‐derived microglia‐like cells with Hyb87

#### Culture of human iPSCs

All human cell protocols used in this study were approved by the Takeda Institutional Ethical Committee and followed the guidelines of the Declaration of Helsinki. Human iPSCs (Clone XCL‐1; XCell Science, Novato, CA, USA) were cultured on laminin‐coated plates in StemFit (Ajinomoto Healthy Supply Co., Inc, Tokyo, Japan) containing penicillin and streptomycin. The cells were passaged every 6–7 days using 0.5 mm EDTA, seeded at a density of 2.0 × 10^4^ cells·well^−1^ on a six‐well plate, and maintained in the presence of 10 μm Y‐27632 (FujiFilm Wako Pure Chemical). The culture medium was replaced with StemFit containing penicillin and streptomycin in the absence of Y‐27632, 24 h after passaging.

#### Differentiation of iPSCs to hematopoietic progenitor cells

iPSC‐derived hematopoietic progenitors (iHPCs) were generated using defined conditions with several modifications to previously published protocols [35, 36]. Briefly, 0.5–1.0 × 10^5^ cells were plated per well in a tissue culture‐treated six‐well plate (day 0). The cells were cultured in 2 mL StemFit containing Y‐27632 for 24 h under normoxic conditions.

Day 1: The medium was changed to basal medium [37] supplemented with 50 ng·mL^−1^ FGF2, 50 ng·mL^−1^ BMP4, 12.5 ng·mL^−1^ activin‐A, 10 μm Y‐27632, and 2 mm LiCl. The cells were then placed under hypoxic cell culture conditions of 5% O_2_ and 5% CO_2_ for 2 days.

Day 3: The cells were further maintained in basal medium supplemented with 50 ng·mL^−1^ each of FGF2 and VEGF under hypoxic conditions.

Day 5: The medium was changed to basal medium containing 50 ng·mL^−1^ FGF2, 50 ng·mL^−1^ VEGF, 50 ng·mL^−1^ TPO, 10 ng·mL^−1^ SCF, 50 ng·mL^−1^ IL‐6, and 10 ng·mL^−1^ IL‐3. The cells were placed under normoxic conditions.

Day 7 and day 9: The medium was changed to the one used on day 5.

#### Differentiation of iHPCs to iMGs

iPS‐derived microglia‐like cells (iMGs) were differentiated from iPSCs via embryoid bodies and HPCs using defined conditions with several modifications to a previously published protocol [37].

Day 11: iHPCs were washed using iMG basal differentiation medium [37]. After centrifugation, the iHPCs were gently suspended in iMG complete differentiation medium containing 25 ng·mL^−1^ M‐CSF, 100 ng·mL^−1^ IL‐34, and 50 ng·mL^−1^ TGF‐β and seeded at a density of 2 × 10^5^ cells per well on 6‐well plates. One milliliter iMG complete differentiation medium was added every 2 days.

Day 23 and day 37: The cells were collected as iMGs and seeded in a 1 : 1 mixture of conditioned medium and iMG complete differentiation medium [37]. The cells were supplemented with 1 mL iMG complete differentiation medium every two days.

The reagents used in this experiment were purchased from Thermo Fisher Scientific (BMP4, activin‐A, VEGF, TPO, IL‐6, SCF, and IL‐3) or PeproTech (FGF2, M‐CSF, and TGF‐β).

#### Treatment of iMGs with Hyb87

iMGs were collected on day 39 and seeded on a 24‐well nontissue culture‐treated plate (Corning) coated with 10 μg·mL^−1^ Hyb87 or control mouse IgG. After 6 h, the cells were harvested for RNA isolation.

### AmpliSeq analysis

AmpliSeq libraries were constructed for iMGs and sequenced in biological triplicates using the Ion Proton platform (Thermo Fisher Scientific) according to the manufacturer's instructions. Briefly, 10 ng of total RNA was reverse transcribed using the SuperScript VILO cDNA Synthesis Kit (Thermo Fisher Scientific), followed by library generation using the Ion AmpliSeq Transcriptome Human Gene Expression Kit. The libraries were diluted to 45 pm and pooled equally with 9–10 individual samples per pool. The pooled libraries were multiplexed and clonally amplified using the Ion Chef System, and subsequently, sequenced on Ion PI chips using an Ion Proton sequencing system. Data were first analyzed using the Torrent Suite, and the ampliSeqRNA analysis plug‐in was used to generate count data. Principal component analysis (PCA) was performed using the ‘prcomp’ function of stats package in r [38]. Tukey's honest significant difference test was conducted to compare the first principal component (PC1) in each group using ‘Tukey HSD’ of stats package in r. Visualization of the scatter plot and boxplot was performed using the ggplot2 package in r. All the AmpliSeq data were deposited in the Gene Expression Omnibus (GEO) repository at http://www.ncbi.nlm.nih.gov/geo (accession number: GSE159333).

### Identification of differentially expressed genes induced by TREM2 activation

The differentially expressed gene (DEGs) representing TREM2 activation (TREM2 DEGs) were identified based on the gene‐wise negative binomial generalized linear model with the quasi‐likelihood method using the edgeR package in r [39, 40]. The criteria for the significance of DEGs were set at false discovery rate‐adjusted (FDR‐adjusted) *P* value < 0.05 and absolute fold change > 1.5. Volcano plots of the DEG analysis were drawn using EnhancedVolcano package in r. TREM2 DEGs were defined as genes common between DEGs from Hyb87 vs PBS and Hyb87 vs control IgG and those with the same direction of expression change in the DEGs.

### Pathway enrichment analysis

Pathway enrichment analyses of TREM2 DEGs were conducted using the Cortellis MetaCore software (Clarivate, Philadelphia, PA, USA) using ‘Enrichment Analysis’ workflow (default setting).

### GSEA

The Religious Order Study and Rush Memory and Aging Project (ROSMAP) data (syn18485175), the single‐cell expression profile of human dorsolateral prefrontal cortex derived from 75 060 cells of 48 individuals [41], was downloaded from AD Knowledge Portal (https://adknowledgeportal.synapse.org/Explore/Studies?Study=syn18485175). The data included quality‐filtered single‐nucleus RNA‐sequenced read counts of 17 926 genes of the human reference genome 38 (GRCh38) and the clustering results of the reads to each cell type. The reads from microglial cells were extracted, and genes with less than three aligned reads were removed, resulting in a total of 13 039 genes. PCA was performed on log‐normalized read counts, and three individuals whose PC1 or second PC2 were outside of more than three standard deviations from that of the other samples were removed. Healthy controls (HC) and patients with AD in the original report [41] were reclassified into HC, MCI, and AD using Mini‐Mental State Examination (MMSE; HC, ≥ 29; MCI, 24–28; AD, ≤ 23) [42] or clinical consensus diagnosis of cognitive status (cogdx: HC, 1; MCI, 2 or 3; AD, 4 or 5) [41]. We compared the expression of genes across HC, MCI, and AD using DESeq2 [43] in Seurat [44]. Finally, we multiplied the −log_10_ *P* value with the sign of the log‐scale fold change (signed *P* value) for gene set enrichment analysis (GSEA). We also quantile‐normalized the read counts on each gene and conducted linear regression on cognitive, MMSE, Consortium to Establish a Registry for AD (CERAD), and Braak scores with covariates of sex, years of education, and age. The CERAD score decreased with the progress of the neuritic plaques, whereas Braak or MMSE scores increased with the deterioration of neurofibrillary tangles or cognitive decline, respectively. To interpret the CERAD GSEA result in the same correlation direction as the MMSE and Braak GSEA results, we calculated the correlations between each gene and CERAD score and converted them to the signed *P* values by reversing the positive and negative directions.

Another single‐nucleus RNA‐seq (snRNA‐seq) dataset generated using ROSMAP data (syn21125841) [27] was analyzed for a replication study. Data included the number of aligned reads per gene in each cell, which were quantified using Cell Ranger Single‐Cell Software Suite (10x Genomics, Pleasanton, CA, USA). Data of 19 individuals which did not overlap with that of ROSMAP syn18485175 data [41] and had the common variant, TREM2, were downloaded from AD Knowledge Portal (https://adknowledgeportal.synapse.org/Explore/Studies?Study=syn21670836). The 19 individual datasets comprised 10 patients with AD and nine HCs. The clustering of the cells was conducted using the R package, Seurat [44]. The cells with a high ratio of mitochondrial reads (> 5%), abnormally high or low number of unique molecular identifiers (< 400 or > 40 000, respectively), or an abnormally high or low number of detected genes (> 9000 or < 200, respectively) were filtered out—resulting in a remainder of 42 087 cells. Next, the read counts for each gene were divided by the total counts in the cell and log‐normalized following multiplication of 10 000. Then, 20 000 features (genes) that were highly variable among the cells were selected using FindVariableFeature in Seurat package [44]. The cells were clustered using shared nearest neighbor modularity optimization using 1–20 PCs after the dimension reduction analysis. Then, the clusters were identified as one of the following cell types via the mean expression level of the marker genes selected by the Allen Institute for Brain Science [45]: *GAD1* (interneuron), *SLC17A7* (excitatory neuron), *TYROBP* (microglia), *AQP4* (astrocyte), *PDGFRA* (oligodendrocyte precursor), *OPALIN* (oligodendrocyte), and *NOSTRIN* (endothelial cell). As a result, 2200 cells were identified as microglia. The reads in the microglia were summed per individual, and genes with less than three reads in total were removed. The gene expression was then compared between AD and HC groups using DESeq2 [43]. The results of each gene were used for GSEA.

Gene set enrichment analysis was conducted between TREM2 DEGs and genes extracted from the above‐mentioned datasets using the GSEA_R package in r. The criterion for the significance of GSEA was set at FDR‐adjusted *q*‐value < 0.05. The datasets generated and analyzed during the current study are available in the GEO repository, https://www.ncbi.nlm.nih.gov/geo/query/acc.cgi?acc=GSE159333.

## Results

### Generation and characterization of agonistic anti‐TREM2 mAb, Hyb87

We attempted to obtain an anti‐TREM2 agonistic Ab. We used hybridoma techniques following the immunization of mice with TREM2‐Fc protein. The culture supernatant of the hybridomas was screened using ELISA to identify the clone secreting suitable mAbs, and our results showed that the anti‐TREM2 Ab clone, Hyb87, bound to human TREM2.

**Fig. 1:**
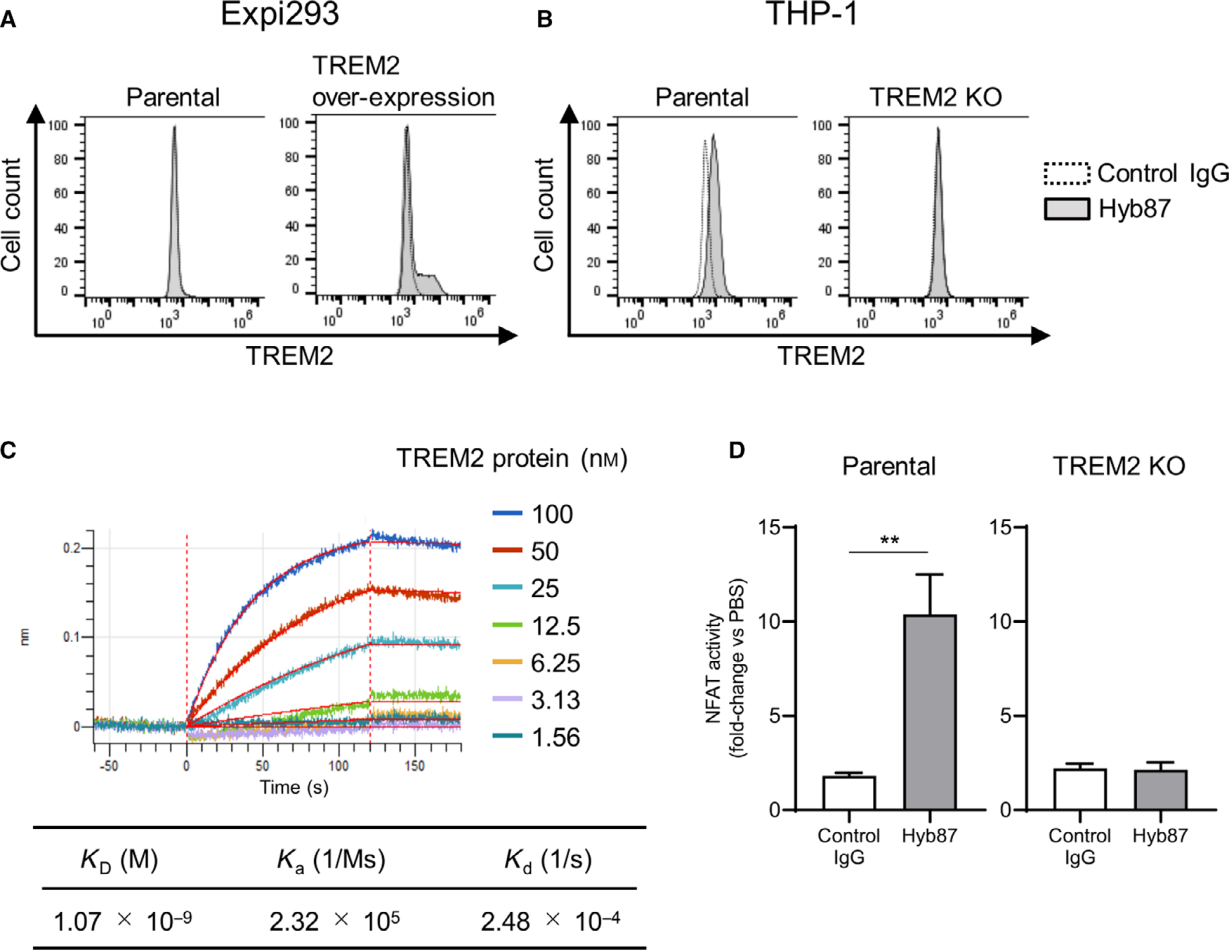
Binding and agonist activities of Hyb87 toward TREM2. (A, B) Binding of Hyb87 to TREM2 in cells was determined using FCM. Cells were incubated with Hyb87, followed by incubation with Alexa488‐labeled anti‐mouse IgG Ab. (A) Parental and human TREM2‐transfected Expi293 cells. (B) THP‐1 cells and *TREM2* KO THP‐1 cells. Control IgG, dashed line; Hyb87, gray histogram. (C) Analysis of Hyb87 affinity to TREM2. BLI was used to determine the affinity of Hyb87 to TREM2. The vertical axis indicates the BLI signal response (nm), and the horizontal line indicates the time after analyte (TREM2 protein) loading. Kinetic parameters were analyzed using a 1 : 1 Langmuir fitting model. Association (*K*_a_) and dissociation (*K*_d_) constants were calculated and used to determine the *K*_D_ value (*K*_d_/*K*_a_). (D) NFAT response of Hyb87. THP‐1 cells and *TREM2* KO THP‐1 cells transiently transfected with a NFAT‐luc plasmid were incubated on a Hyb87‐coated plate. Data points represent the mean + SD of values acquired in triplicate. ** for *P* value < 0.005 vs control IgG‐treated group by Student's *t*‐test. All data are representative of at least two independent experiments.

We further characterized Hyb87 using multiple assays. First, we tested the binding of Hyb87 to TREM2, both exogenously and endogenously expressed in cells. We prepared Expi293 cells transfected with the human TREM2 expression plasmid. Although the fluorescence intensity of the control IgG did not increase in parental or human TREM2‐overexpressing Expi293 cells in FCM that of Hyb87 did when human TREM2 was expressed (Fig. 1A). We further confirmed the binding of Hyb87 to endogenous TREM2. We used human monocytic THP‐1 cells for TREM2 expression [46]. As shown in Fig. 1B, fluorescence intensity was detected in THP‐1 cells after adding Hyb87. To further verify the specific binding of Hyb87, we established *TREM2* KO THP‐1 cells and observed that Hyb87 binding was lost in these cells (Fig. 1B). These results indicated that Hyb87 bound specifically to human TREM2 on the cell surface. The affinity of Hyb87 to TREM2 was then determined using BLI. The Ab was immobilized onto the sensor chip as a ligand, and TREM2‐His was applied as an analyte. According to an analysis using a Langmuir fitting model, the *K*_D_ value of Hyb87 to human TREM2 was 1.07 × 10^−9^ m. The *K*_a_ of the Ab was determined to be 2.32 × 10^5^ (1/Ms ), and the *K*_d_ was 2.48 × 10^−4^ (1/s; Fig. 1C).

Next, we investigated whether Hyb87 activated TREM2. A previous study reported that TREM2 activation increases intracellular Ca^2+^ concentration and can be detected using the NFAT reporter [47, 48]. Hence, we used the NFAT reporter system in this study. THP‐1 cells were transiently transfected with the reporter plasmid and then allowed to react with Hyb87. Compared to the PBS‐treated group, Hyb87 increased NFAT activity by 10.4‐fold, whereas the increase by control IgG was 1.8‐fold (Fig. 1D). This indicated that Hyb87 activated TREM2. Furthermore, we observed a slight increase in NFAT reporter activity by control IgG. We believe that the increase in NFAT activity by control IgG was a consequence of the binding of the control IgG to the Fc receptor [49]. Meanwhile, NFAT reporter activity by Hyb87 was not significantly different than that by control IgG in *TREM2* KO THP‐1 cells (Fig. 1D), suggesting that NFAT activity by Hyb87 in the THP‐1 cells was mediated through TREM2.

### Isolation of TREM2 DEGs from iMG

**Fig. 2:**
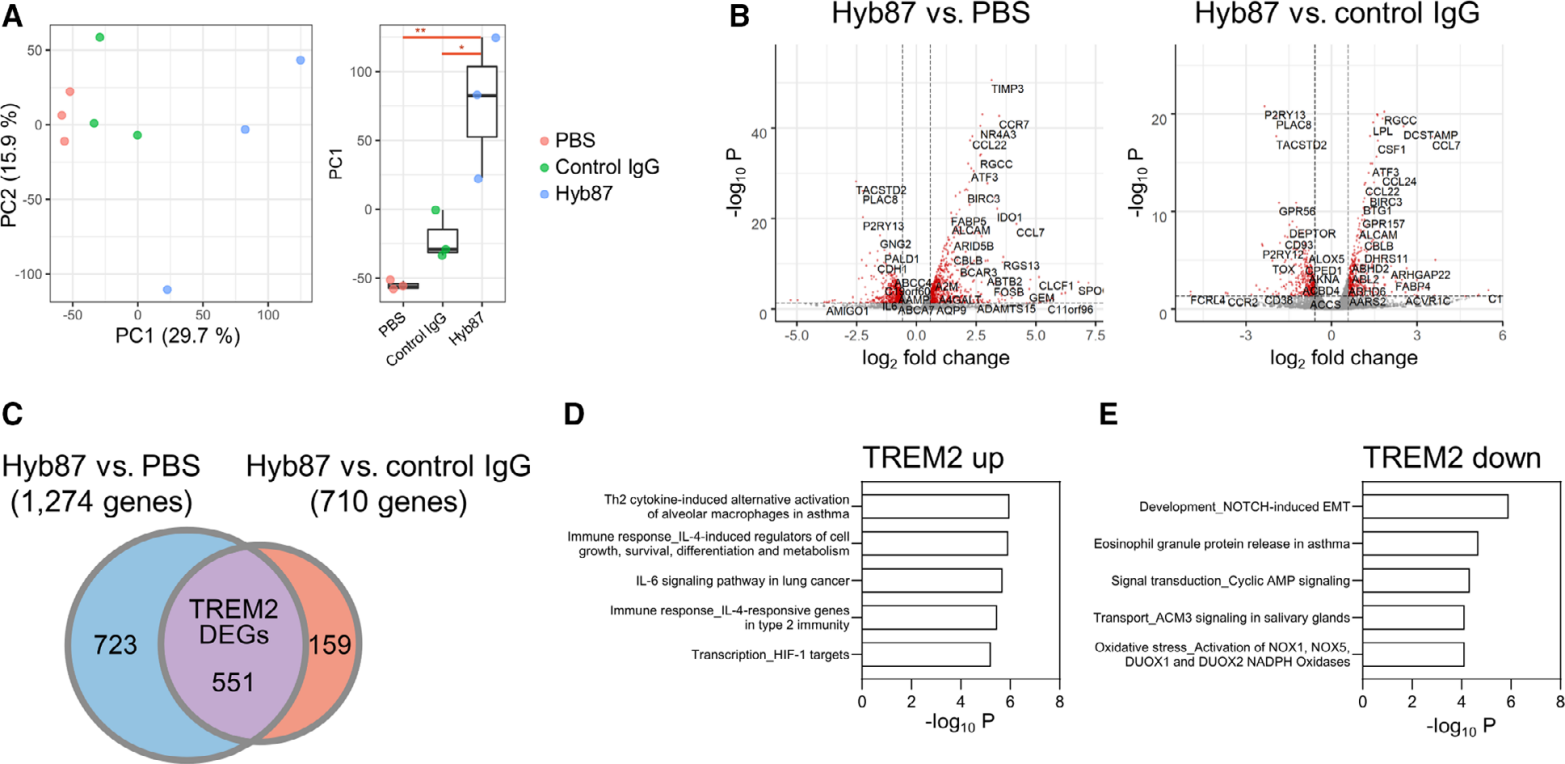
Identification of TREM2 DEGs from iMG. (A) PCA and boxplot analysis of the PC1 distribution of the iMG transcriptome data from PBS, control IgG, and Hyb87 treatment groups. Each group had three replicates. Percentages with each axis represent the variance of the data captured by each PC. * or ** for boxplot indicates *P* value calculated using Tukey's honest significant difference test between groups. * for *P* value < 0.05 and ** for *P* value < 0.01. (B) Volcano plots depicting the results of differential gene expression analysis between Hyb87 and PBS treatment or between Hyb87 and control IgG treatment. The criteria for significance were set at FDR‐adjusted *P* value < 0.05 and absolute fold change > 1.5. DEGs that satisfy the criteria are shown in red. (C) Venn diagram showing TREM2 DEGs. Genes that overlapped between those from Hyb87 treatment vs PBS and from Hyb87 treatment vs control IgG treatment were defined as TREM2 DEGs. Considering the direction of expression alteration, the TREM2 DEGs comprised genes, the expression of which changed in the same direction in Hyb87 treatment (vs PBS and vs control IgG). (D, E) Top five significant pathways in pathway enrichment analysis using TREM2 up (D) and TREM2 down (E).

Next, we investigated how TREM2 activation changed in the microglia of patients with AD. We assessed the changes in the expression of genes downstream of TREM2 to understand TREM2 activation in AD. We consistently used human data, as significant differences in the transcriptional signatures of human AD and the 5xFAD mouse model have been reported [27]. To determine gene expression changes downstream of TREM2, iMG cells were treated with Hyb87, PBS, or control IgG and subjected to transcriptome analysis using the AmpliSeq approach. In PCA of the iMG AmpliSeq data, the variance of PC1 and PC2 was found to be 29.7% and 15.9%, respectively (Fig. 2A). In the PC1 that explained the maximum variabilities, Hyb87 differed significantly from the PBS control and IgG, indicating that Hyb87‐induced changes in the expression of specific genes could be detected. Next, we isolated DEGs in response to Hyb87 treatment (vs PBS or vs control IgG; Table S1). We observed significant changes in gene expression after Hyb87 treatment as per the following criteria: FDR‐adjusted *P* value < 0.05 and absolute fold change > 1.5, which involved 1274 genes in Hyb87 vs PBS and 710 genes in Hyb87 vs control IgG (Fig. 2B,C, and Table S1). In contrast, the DEGs from control IgG (vs PBS) consisted of as few as 81 genes (Table S1). To exclude gene expression changes due to IgG via Fc receptors, we attempted to define TREM2 DEGs that overlapped in the direction of altered expression between Hyb87 vs PBS and Hyb87 vs control IgG. This allowed us to define TREM2 activation for 551 genes (Fig. 2C and Table S2) consisting of 300 upregulated (TREM2 up) and 251 downregulated (TREM2 down) DEGs (Fig. 2B and Table S1). The TREM2 DEGs were applied to pathway enrichment analysis using MetaCore. TREM2 up showed enrichment in ‘Immune response_TSLP signaling’ (*P* = 3.0E‐7), ‘Th2 cytokine‐induced alternative activation of alveolar macrophages in asthma’ (*P* = 1.1E‐06), and ‘Immune response_IL‐4‐induced regulators of cell growth, survival, differentiation, and metabolism’ (*P* = 1.2E‐06). TREM2 down was associated with ‘Development_NOTCH‐induced EMT’ (*P* = 1.3E‐06), ‘Eosinophil granule protein release in asthma’ (*P* = 2.0E‐05), and ‘Signal transduction_Cyclic AMP signaling’ (*P* = 4.6E‐05; Fig. 2D,E, and Table S3).

### Reduction in TREM2 activation with disease progression in patients with AD

**Fig. 3:**
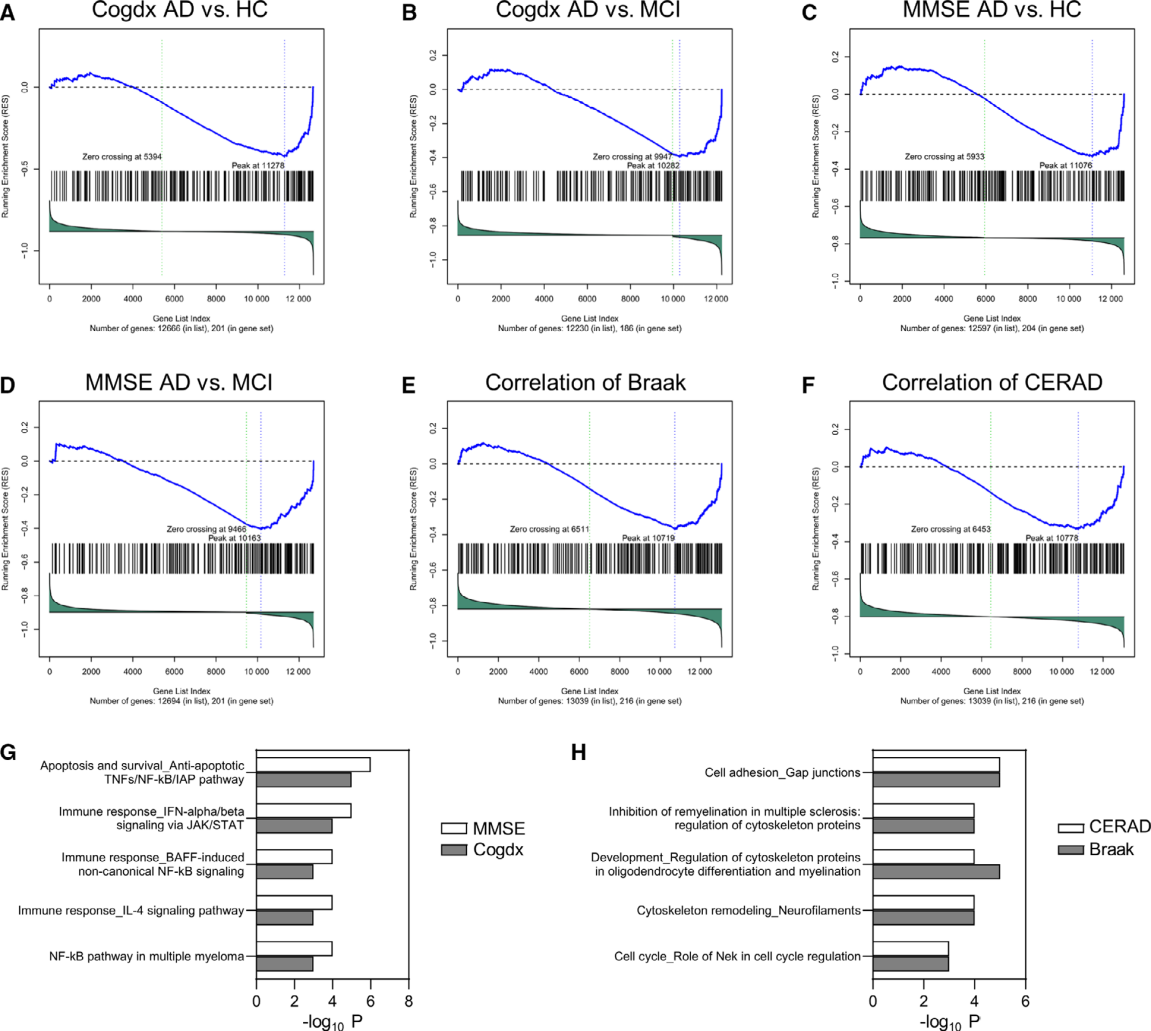
Reduction in TREM2 activation in AD microglia. (A–F) GSEA enrichment plots of ROSMAP syn18485175 data (samples from 45 individuals were used: six HC, 17 MCI, and 22 AD individuals classified by MMSE or 13 HC, 10 MCI, and 21 AD individuals classified by cogdx) that were significantly enriched. The vertical axis shows all genes arranged in the order of signed *P* value. The horizontal axis indicates the enrichment score of each gene. (G, H) Top five significant pathways in pathway enrichment analysis using genes enriched in GSEA between TREM2 up and DEGs in AD (vs HC) (G) and between TREM2 up and Braak or CERAD (H).

Next, we investigated how TREM2 is activated in patients with AD by comparing the TREM2 DEGs with the transcriptome data of microglia from these patients. We used GSEA to determine statistically significant and consistent differences between gene sets from two biological states [50, 51]. We separately used TREM2 up (300 upregulated genes) and TREM2 down (251 downregulated genes) to clarify the direction of gene expression regulated by TREM2 activation. We selected snRNA‐seq data of patients with AD for comparison, for the following reasons: The microglial population that expresses TREM2 exclusively consisted of only 5–10% brain cells; TREM2 DEGs were from iMG, but not limited to microglia. Therefore, single‐cell‐derived RNA‐seq data were ideal for our study, as gene expression changes in microglia may not be detected in the bulk RNA‐seq data of tissues consisting of a mixed cell population. We performed GSEA using TREM2 DEGs in two ways: (a) statistical analysis using DEGs among divided populations and (b) correlation analysis using AD disease scores obtained from CERAD, Braak, and MMSE. First, we excluded three out of the 48 individuals using PCA, which suggested low data quality, and then, we classified 45 individuals into three populations of HC, MCI, or AD using two clinical scores; MMSE of HC (≥ 29), MCI (24–28), and AD (≤ 23), or cogdx of HC (value 1), MCI (value 2, 3), and AD (value 4, 5), leading to six HC, 17 MCI, and 22 AD individuals determined using MMSE, or 13 HC, 10 MCI, and 21 AD individuals determined using cogdx (Table S4), after assessing the quality using PCA and excluding one individual from cogdx classification due to the possibility of other diseases. Next, we calculated the statistical values and signed *P* value in each population by comparing HC with MCI, HC with AD, or MCI with AD. GSEA was then conducted using these signed *P* values as the score on the side of the molecular profile data and TREM2 up or down as the data on the dataset side. Intriguingly, we observed that TREM2 up was significantly lower in AD microglia than in HC or MCI microglia when each population was defined using both MMSE and cogdx (Fig. 3A–D and Table 1). Furthermore, no significant enrichment was observed when we compared HC and MCI microglia. These findings clearly showed that TREM2 activation was lost in AD. However, TREM2 down was significantly associated only with cogdx AD vs MCI, which meant that TREM2 down was significantly lower in AD microglia than in MCI microglia. We speculated that the fluctuation range of the fold change was significantly lower in TREM2 down than in TREM2 up, as absolute values of mean fold change were 1.09 in TREM2 up and 0.98 in TREM2 down, with *P* = 0.03. Second, the relationship between CERAD‐, Braak‐, or MMSE‐correlated microglia genes and TREM2 DEGs (TREM2 up or TREM2 down) was investigated using GSEA to determine whether TREM2 activation changed with the progression of AD. Toward this, we used GSEA to compare TREM2 DEGs with genes that correlated with CERAD, Braak, or MMSE scores, which represented the semiquantitative measure of neuritic plaques, neurofibrillary tangles, and cognitive function, respectively. TREM2 up showed significant correlation with CERAD (FDR *q*‐value = 0.009) and Braak‐correlated genes (FDR *q*‐value = 0), but not with MMSE‐correlated genes (FDR *q*‐value = 0.562; Fig. 3E,F and Table 2). Furthermore, TREM2 down was not significantly associated with any of the scores tested. Collectively, these results suggested that TREM2 activation decreased with the progression of AD pathology.

**Table 1:** Correlation between TREM DEGs and AD vs HC vs MCI microglia using GSEA. GSEA results between TREM DEGs and signed *P* value of AD vs HC, MCI vs HC, and AD vs MCI in microglia of ROSMAP syn18485175 data.

Dataset	TREM2 DEG	NES	FDR *q*‐value^a^
Cogdx AD vs HC	TREM2 up	−1.638	0
Cogdx AD vs MCI	TREM2 up	−1.565	0
MMSE AD vs HC	TREM2 up	−1.350	0.001
MMSE AD vs MCI	TREM2 up	−1.557	0.002
Cogdx AD vs MCI	TREM2 down	1.342	0.019
MMSE AD vs MCI	TREM2 down	1.191	0.085
Cogdx MCI vs HC	TREM2 down	−1.117	0.265
Cogdx AD vs HC	TREM2 down	1.099	0.477
MMSE AD vs HC	TREM2 down	1.087	0.514
MMSE MCI vs HC	TREM2 up	1.111	0.663
Cogdx MCI vs HC	TREM2 up	0.797	0.811
MMSE MCI vs HC	TREM2 down	0.823	0.903

^a^ FDR *q*‐value indicates FDR‐adjusted *P* value of GSEA.

**Table 2:** Correlation between TREM2 DEGs and clinical scores of AD using GSEA. GSEA results between TREM DEGs and clinical score of AD of ROSMAP syn18485175 data.

Dataset	TREM2 DEG	NES	FDR *q*‐value^a^
Correlation of Braak	TREM2 Up	−1.527	0
Correlation of CERAD	TREM2 Up	−1.458	0.009
Correlation of CERAD	TREM2 Down	−0.957	0.439
Correlation of MMSE	TREM2 Down	0.975	0.472
Correlation of MMSE	TREM2 Up	1.043	0.562
Correlation of Braak	TREM2 Down	0.849	0.788

^a^ FDR *q*‐value indicates FDR‐adjusted *P* value of GSEA.

Based on these findings, genes enriched in GSEA between TREM2 up and the core enrichment genes in AD (vs HC) that regulated TREM2 activation in AD were applied to pathway enrichment using MetaCore. Interestingly, the following were selected as the common pathways: ‘Apoptosis and survival_anti‐apoptotic TNFs/NF‐kB/IAP pathway’ (*P* = 1.0E‐6 on cogdx; *P* = 2.9E‐7 on MMSE), ‘Immune response_IFN‐alpha/beta signaling via JAK/STAT’ (*P* = 3.4E‐5 on cogdx; *P* = 1.0E‐5 on MMSE), and ‘Immune response_BAFF‐induced noncanonical NF‐kB signaling’ (*P* = 1.1E‐4 on cogdx; *P* = 4.3E‐5 on MMSE). In contrast, pathway enrichment analysis with genes from GSEA and Braak or CERAD indicated ‘Development_regulation of cytoskeleton proteins in oligodendrocyte differentiation and myelination’ (*P* = 1.8E‐6 on Braak; *P* = 5.6E‐5 on CERAD), ‘Inhibition of remyelination in multiple sclerosis: regulation of cytoskeleton proteins’ (*P* = 1.6E‐5 on Braak; *P* = 1.7E‐5 on CERAD), and ‘Cell adhesion_gap junctions’ (*P* = 3.3E‐6 on Braak; *P* = 3.6E‐6 on CERAD) as the common pathways (Fig. 3G,H, and Table S5).

We investigated whether TREM2 expression differed between AD, MCI, and HC, or whether TREM2 expression correlated with CERAD or Braak scores. Our results showed that TREM2 expression did not differ significantly between AD, MCI, and HC and did not show any correlation with the scores (FDR‐adjusted *P* value > 0.85; Tables S6 and S7).

We analyzed another snRNA‐seq dataset, ROSMAP data (syn21125841) [27], to replicate our observations. Nineteen individuals were classified into three populations of HC, MCI, or AD using two clinical scores: MMSE or cogdx. Three HC, five MCI, and 11 AD individuals were determined using MMSE, or nine HC, and 10 AD individuals were determined using cogdx. (Table S8). We calculated the signed *P* value from the snRNA‐seq data between HC and AD microglia and performed GSEA using TREM2 up for replication. Our results showed that TREM2 up was significantly lower in AD microglia than that in HC microglia (Fig. S1A,B).

## Discussion

In this study, we generated an agonistic anti‐TREM2 mAb, Hyb87, and utilized it as a tool for activating TREM2. Further, we defined TREM2 activation by assessing changes in the expression of downstream genes. Previous studies used anti‐TREM2 Ab as a TREM2 activation tool in various types of cells, including the microglia, dendritic cells, macrophages, osteoclasts, and astrocytes [19, 20, 47, 52, 53, 54, 55, 56, 57, 58]. However, in these studies, the Abs were used to visualize signals and/or assess the cellular functions of activated TREM2 in terms of intracellular Ca^2+^ mobilization, ERK phosphorylation, SYK phosphorylation, apoptotic cell death, the formation of TRACP^+^ osteoclasts, migration of osteoclasts, pro‐ and anti‐inflammatory responses, phagocytosis, cell survival, migration, and amyloidogenesis, but not for defining TREM2 activation, as performed in this study.

We used Hyb87 to activate TREM2 in iMG cells and identified TREM2 DEGs after comparison with control IgG stimulation (Fig. 2A–C). We observed a slight increase in the NFAT signal by the control IgG (Fig. 1D). The control IgG‐mediated responses were possibly due to Fc receptor engagement. A previous report showed that NFAT, as well as other transcription factors, transmit signals downstream of the Fc receptor [49]. Fc receptor engagement and TREM2 increase the expression levels of some of the cell surface molecules, including CD86, CD40, or CCR7 [47]. The fact that TREM2 induces signaling downstream of the Fc receptor suggests that the DEGs shared between TREM2 and control IgG treatment, which were not included in our TREM2 DEGs, may primarily be a part of TREM2 DEGs. Wang *et al*. demonstrated that mutations abolishing IgG binding to the Fc receptor in the agonistic anti‐TREM2 mAb marginally affect its prosurvival effect on macrophages, suggesting that the Ab‐mediated TREM2 activation is largely independent of the IgG‐mediated cross‐linking of Fc receptors [20]. This finding strongly supports our definition of TREM2 DEGs.

Pathway enrichment analysis of TREM2 up showed type 2 immune response as represented by the Th2 type cytokine pathways, including TSLP and IL‐4 (Fig. 2D and Table S3). Our findings agree with those of previous studies, wherein TREM2 was reported to enhance Th2 cytokine production [33, 54, 59]. Th2 cytokines also inhibit Aβ_1–42_‐induced pro‐inflammatory cytokines, including IL‐6 and IL‐1β, in microglia and THP‐1 cells [60]. IL‐4 induces the uptake and degradation of Aβ_1–42_ [61]. Intracerebral microinjection of IL‐4 and IL‐13 reduces Aβ accumulation in APP23 mice via microglial activation [62]. Thus, the TREM2 signal may modulate Th2 type cytokine pathways in Aβ clearance.

We successfully showed low TREM2 activation in microglia of patients with AD and that TREM2 activation decreased with the progression of neuritic plaques and neurofibrillary tangles, represented by CERAD and Braak scores, respectively. This indicated TREM2‐mediated regulation of AD pathology via controlling microglial functions and corroborated a previous finding that the TREM2 loss‐of‐function variant R47H is associated with increased density of neuritic plaques and neurofibrillary tangles in multiple brain regions [13]. R47H is associated with amyloid compaction and increased tau hyperphosphorylation around amyloid deposits [63]. It is noteworthy that TREM2 activation was not associated with the cognition score, MMSE, in our analysis. A report examining the association of MMSE and R47H variant revealed no significant difference in the annual rate of MMSE decline between patients with AD carrying a TREM2 common variant and an R47H variant, although the number of participants was small [64]. Further, the R47H risk variant did not significantly affect cognitive performance in a longitudinal study [65]. Out of the 45 donors in the dataset that we used in the evaluation study (Fig. 3), 29 were registered as harboring a TREM2 common variant. On the other hand, TREM2 sequences of the remaining 16 donors were not registered. Therefore, we cannot rule out the possibility that the 16 donors possessed the R47H variant that affected our analysis in elucidating TREM2 activation. However, our replication study recapitulated the results of the evaluation study (Fig. S1), and the dataset used in the replication study comprised TREM2 common variant carriers alone. This indicates that TREM2 signal is low in microglia of patients with AD regardless of the R47H variant.

We speculated that lower TREM2 activation in the microglia of patients with AD could be caused by proteases. Several proteases, including ADAM10, ADAM17, or meprin β, are known to reduce the levels of membrane‐bound TREM2 [28, 29, 66]. The ADAM17‐mediated shedding of the membrane‐bound TREM2 is triggered by pro‐inflammatory stimulation, such as lipopolysaccharide, TNF‐α, or IFN‐γ [67]. The reduction of the membrane‐bound TREM2 levels by ADAM10 or meprin β has been shown to inhibit TREM2‐mediated phagocytosis [66]. Another study reported that γ‐secretase degrades the C‐terminal fragment of TREM2 and that impaired γ‐secretase activity leads to an accumulation of the C‐terminal fragment of TREM2, thereby trapping its adaptor protein—TYROBP to reduce TREM2 signaling [68, 69].

Our GSEA data showed that TREM2 activation was not significantly different in MCI (vs HC), suggesting that TREM2 activation could be maintained in the microglia of patients with MCI. As discussed earlier, the function of TREM2 could be affected by proteases that behave differently in MCI and AD microenvironments. Interestingly, a previous report showed that the levels of active form of γ‐secretase activating protein reduced in the frontal cortex of severe AD subjects, but not in that of MCI subjects [70]. This difference in protease regulation could contribute to the varying amounts of membrane‐bound TREM2 capable of transmitting TREM2 signal in the microglia of patients with MCI and AD. Previously, Jiang *et al*. [71] reported that TREM2 failed to improve Aβ pathology when the lentiviral overexpression of TREM2 was tried in 18‐month‐old APP/PS1 mice, whereas its overexpression improved Aβ pathology in 7‐month‐old APP/PS1 mice [16]. This suggests that the TREM2 signal is no longer viable once AD has been established. However, further studies are required to understand how TREM2 activation changes with disease progression.

In the present study, the TREM2 expression levels alone showed no correlation with the clinical scores from the snRNA‐seq data used. Although some previous studies reported TREM2 expression changes in AD brains due to aging [21, 22, 23, 24, 25, 26, 27], the direction of alteration of TREM2 expression levels is not unilateral. For example, in the hippocampus of patients with AD, TREM2 mRNA has been reported to be higher than that of controls [21], but different groups exhibit either downregulation of TREM2 or no alteration at protein and/or mRNA levels [22, 23, 24]. Although extensive efforts are required to address this discrepancy, some reasons for it may be as follows. TREM2 is mainly expressed in microglia in the brain. Differences in the population size of microglia could lead to changes in the relative expression of TREM2. Additionally, the use of different internal controls for normalization may lead to difficulty in the interpretation of TREM2 expression. The expression level of a molecule is typically normalized with an internal control, such as *GAPDH*, *ACTB*, or *HPRT*. In practice, TREM2 mRNA has been differently normalized in previous studies: with *GAPDH* [22, 24], a combination of *ACTB* and *HPRT* [21], or a combination of *GAPDH* and *HPRT* [25]. Moreover, patient demographics may have been different in those studies or sample storage conditions could have affected sample quality. However, none of those studies reported an association of TREM2 expression with its activation.

In previous reports, the sTREM2 level was investigated in CSF to determine its association with AD. Interestingly, a few studies revealed that sTREM2 level in the CSF of patients with AD is associated with hallmarks of AD, such as Aβ_1–42_ [72], total tau, and phosphorylated tau in CSF [30, 31, 32]. However, whether sTREM2 levels reflect TREM2 activation remains unknown. The levels of TREM2 proteases, ADAM10 and ADAM17, are altered in AD CSF or brain [73, 74, 75, 76]. Hence, the usability of sTREM2 for TREM2 activation warrants further investigation.

The brain snRNA‐seq data of 5xFAD mice and human patients with AD elucidated distinct AD gene signatures between human and mouse microglia [27]. Notably, the human brain shows more qualitative changes in the number of microglia than that of mice, and the signature of human microglia in AD is different from that of disease‐associated microglia in the 5xFAD model. This finding prompted us to use human cell and tissue data instead of mouse data. The study reported higher expression of the transcription factor IRF8 in AD, indicating that IRF8 may be a major driver of human microglia signature in AD. In our study, *IRF8* was significantly downregulated in response to Hyb87 vs PBS (FDR‐adjusted *P* = 2.6E‐5 and log_2_ fold change = −0.54 for IRF8), indicating that Hyb87 could reverse the IRF8 signature in human AD microglia. Pathway enrichment data with genes enriched in GSEA between TREM2 up and core enrichment genes in AD (vs HC) yielded the anti‐apoptotic pathway as the top hit. This corroborated anti‐apoptotic function of TREM2 in microglia from 5xFAD mice crossed with *TREM2* KO mice [6] or in microglial BV‐2 cells [77]. In addition to the pathway enrichment data, some of the genes were enriched in GSEA function in microglia and could control microglial fate downstream of TREM2. Genes such as heparin‐binding epidermal growth factor‐like growth factor (*HBEGF*), baculoviral IAP repeat‐containing 3 (*BIRC3*), BTG anti‐proliferation factor 1 (*BTG1*), CD300 molecule‐like family member B (*CD300LB*), IL‐3 receptor subunit alpha (*IL‐3RA*), and platelet‐derived growth factor‐alpha (*PDGFA*) were upregulated in response to Hyb87 treatment. Thus, TREM2 activation might efficiently combat AD by orchestrating the expression of the microglial genes involved in microglial fate. HBEGF is a potent stimulator of cell proliferation and migration and plays diverse physiological roles, including wound healing and cardiac development [78]. Previous reports suggest the involvement of HBEGF in microglia and AD. HBEGF signaling stimulates cell proliferation and phagocytosis of microglia [79]. Furthermore, a trans‐ethnic meta‐analysis of a genome‐wide association study identified an intergenic single nucleotide polymorphism between *HBEGF* and *PFDN1* as an AD susceptibility locus [80]. Study of *Hbgef* KO also showed that HBEGF regulates Aβ_1–42_ and phosphorylated tau levels [81], as well as neurogenesis and cognitive function [82]. BIRC3, also known as cIAP2, is an IAP family protein that regulates apoptosis by blocking caspase activation and inflammation via innate immune receptors [83]. In microglia, BIRC3 acts as a switch between pro‐inflammatory activation and cell death by regulating caspase‐3 processing [84]. BIRC3 confers resistance to cell death in microglia subjected to chronic inflammatory stress [85]. BTG1 plays an important role in cell growth and differentiation [86]. In microglia, it regulates microglial apoptosis by functioning as a sensitizer in activation‐induced cell death [87]. CD300LB is an activator of the CD300 family of myeloid immunoglobulin receptors. Interestingly, CD300LB shares similarity with TREM2, as it associates with DAP12 and recognizes phosphatidylserine as a ligand on the outer plasma membrane of apoptotic cells, thereby regulating the efferocytosis and phagocytosis of apoptotic cells [88, 89]. Induction of CD300LB by TREM2 activation indicated that TREM2 may mobilize functionally similar molecules to enhance its effects. IL‐3RA transduces the IL‐3 signal. Different groups have demonstrated IL‐3 induced microglial proliferation using a neutralizing anti‐IL‐3 Ab [90] and its dependence on JAK2 [91]. PDGFA plays physiological roles via PDGFR‐α during gastrulation and in the development of multiple tissues, including the lungs, intestine, and central nervous system [92]. A recent report revealed that PDGFA also exerts proliferative effects on the microglial cells [93]. To validate our speculation, further research is needed for elucidation of the role of TREM2 in AD microglia.

In this study, we focused on microglial data. Interestingly, although peripheral monocytes do not generally express TREM2, its expression was upregulated in monocytes from patients with AD [47, 94], thereby potentially corroborating the upregulated TREM2 expression in the microglia of patients with AD [26, 27]. Monocyte population and functions have also been reported to change in peripheral blood of patients with AD. Previous studies on peripheral immune cells of patients with AD demonstrated an increase in the population of activated monocytes [95, 96], suggesting its pro‐inflammatory nature [96, 97]. If such monocytic changes correlate with TREM2 activation status in the microglia, they may act as a potential biomarker for microglial TREM2 activation in AD, which can be determined in a less invasive approach. However, further investigations are required to validate these speculations.

## Conclusions

Our results show that low TREM2 activation in patients with AD and the agonistic anti‐TREM2 mAb may render microglia resistant to AD progression. Furthermore, our study outcomes provide evidence that agonistic anti‐TREM2 mAb is a powerful tool to identify TREM2 activation in the microglia of patients with AD.

## Conflict of interest

The work was funded by Takeda Pharmaceutical Company, and the authors of the publication were employees of Takeda Pharmaceutical Company at the time the research was conducted.

## Author contributions

YO identified the DEGs based on the studies on iMGs, performed GSEA, conducted pathway enrichment analysis, and wrote the manuscript. HS screened the anti‐TREM2 mAbs and measured Hyb87 activity using the NFAT assay. SM screened the anti‐TREM2 mAbs and determined Hyb87 specificity in FCM. MI conducted the iMG experiment and wrote the manuscript. JL processed the ROSMAP syn18485175 data and wrote the manuscript. DD generated the anti‐TREM2 mAbs and wrote the manuscript. AB supervised Ab generation. YZ and KY processed the ROSMAP syn21125841 data and wrote the manuscript. TA supervised the bioinformatic analyses. SS designed the studies, measured Hyb87 affinity, and critically revised the manuscript. All authors have read and approved the final version of the manuscript.

## Supporting information


**Fig. S1.** Reduction in TREM2 activation in AD microglia (replication study). (A) GSEA normalized enrichment scores (NES) and FDR *q*‐value. (B) GSEA enrichment plots of the replication data set (ROSMAP syn21125841 data: samples from 19 individuals were classified into three HC, five MCI, and 11 AD by MMSE, or nine HC and 10 AD by cogdx) that was significantly enriched. The vertical axis shows all genes arranged in the order of signed *P* value. The horizontal axis indicates the enrichment score of each gene.

**Table. S1.** DEGs of Hyb87 (vs PBS or control IgG).

**Table. S2.** TREM2 DEGs.

**Table. S3.** Pathway enrichment analysis of TREM2 DEGs.

**Table. S4.** Clinical profiles of donors from ROSMAP syn18485175 data.

**Table. S5.** Pathway enrichment analysis of genes enriched in GSEA between TREM2 up and AD microglia.

**Table. S6.** Comparison of TREM2 expression levels between AD vs HC vs MCI microglia.

**Table. S7.** Correlation between TREM2 expression levels and Braak, CERAD, and MMSE scores in AD microglia.

**Table. S8.** Clinical profiles of donors from ROSMAP syn21125841 data.

## Data Availability

All AmpliSeq data obtained in this study were deposited in the Gene Expression Omnibus (GEO) repository at http://www.ncbi.nlm.nih.gov/geo (accession number: GSE159333). The Religious Order Study and Rush Memory and Aging Project (ROSMAP) data used here (syn18485175 and syn21125841) are available at AD Knowledge Portal (https://adknowledgeportal.synapse.org/Explore/Studies?Study=syn18485175 and https://adknowledgeportal.synapse.org/Explore/Studies?Study=syn21670836).
