# Supplementary figures and images for: Reduced TREM2 activation in microglia of patients with Alzheimer's disease
Source: FEBS Open Bio. 2021 Sep 28;11(11):3063–80. doi: 10.1002/2211-5463.13300 (PMC8564098; doi:10.1002/2211-5463.13300)

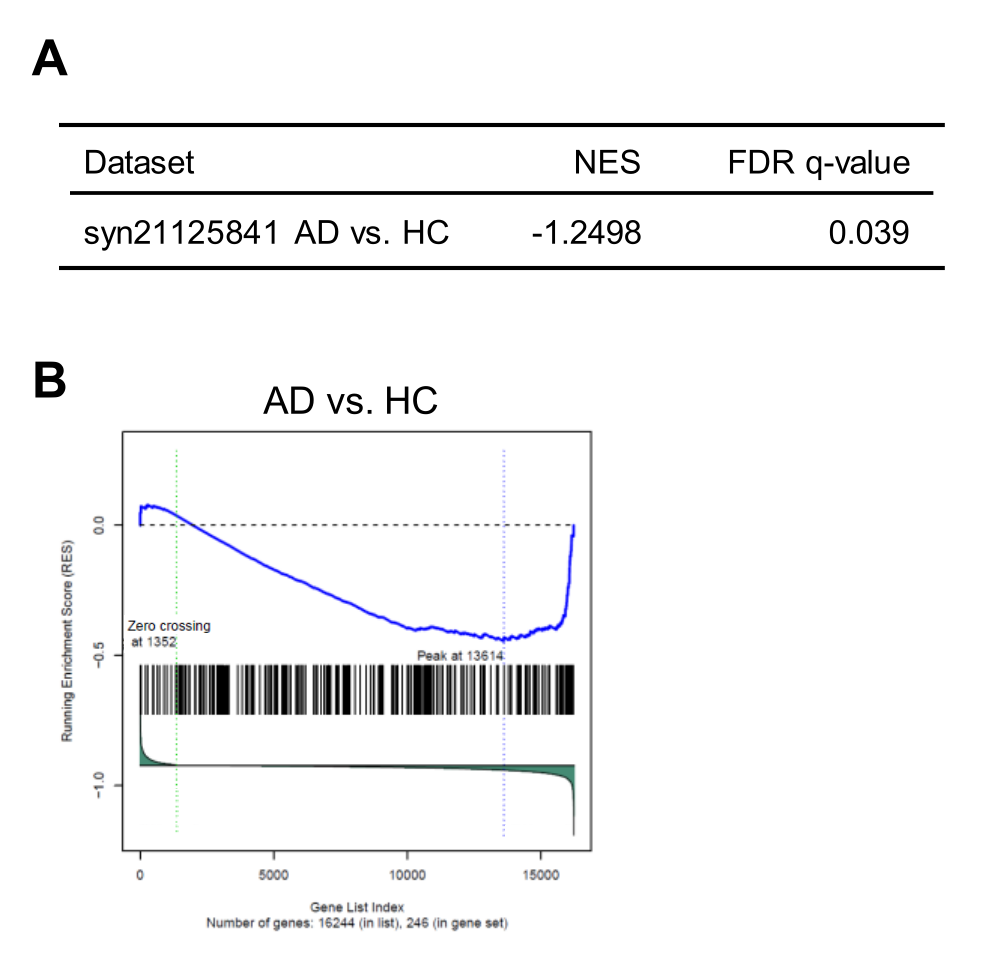

Supplement: Supplementary file 1 — Fig. S1. Reduction in TREM2 activation in AD microglia (replication study). (A) GSEA normalized enrichment scores (NES) and FDR q‐value. (B) GSEA enrichment plots of the replication data set (ROSMAP syn21125841 data: samples from 19 individuals were classified into three HC, five MCI, and 11 AD by MMSE, or nine HC and 10 AD by cogdx) that was significantly enriched. The vertical axis shows all genes arranged in the order of signed P value. The horizontal axis indicates the enrichment score of each gene. [file FEB4-11-3063-s004.tif]
